# Biomedicine, self and society: An agenda for collaboration and engagement

**DOI:** 10.12688/wellcomeopenres.15043.1

**Published:** 2019-01-23

**Authors:** Martyn Pickersgill, Sarah Chan, Gill Haddow, Graeme Laurie, Devi Sridhar, Steve Sturdy, Sarah Cunningham-Burley

**Affiliations:** 1 Centre for Biomedicine, Self and Society, University of Edinburgh, Edinburgh, UK

**Keywords:** Social Science, Humanities, Biomedicine, Disease, Bodies, Global Health, Law, Engagement

## Abstract

The commitment of massive resources – financial, social, organisational, and human – drives developments in biomedicine. Fundamental transformations in the generation and application of knowledge are challenging our understandings and experiences of health, illness, and disease as well as the organisation of research and care. Coupled with the accelerated pace of change, it is pressing that we build authentic collaborations across and between the biomedical sciences, humanities and social sciences, and wider society. It is only in this way that we can ask and answer the penetrating questions that will shape improvements in human health now and in the decades ahead. We delineate the need for such commitments across five key areas of human and societal experience that impact on and are impacted by developments in biomedicine: disease; bodies; global movements and institutions; law; and, science-society engagements. Interactions between ideas, researchers, and communities across and within these domains can provide a way into creating the new knowledges, methods, and partnerships we believe are essential if the promises of biomedicine are to be realised.

## Disclaimer

The views expressed in this article are those of the author(s). Publication in Wellcome Open Research does not imply endorsement by Wellcome.

## Introduction

Promise of new knowledge of health and disease and new means of preventing and treating illness—as well as the economic and commercial benefits such innovations might bring—animates biomedicine from top to bottom: from government policy makers and public and private funders, to individual doctors and researchers. In short, promise underpins the massive commitment of resources—financial, social, organisational, human—that drives biomedicine ( Brown & Michael, 2003). With such enormous resources in play, it is unsurprising that developments in biomedicine are having a profound impact across many areas of human life and experience, the dynamics of which need urgent and rigorous attention ( Rose, 2007). What biomedicine delivers is not always the same as what it promises: it is often only with the wisdom of hindsight that we are able to separate promise from what seems like inevitable hype ( Feiler *et al*., 2017). An enterprise as vast as biomedicine is bound up with multiple, and often conflicting institutions and interests, which pull it in different directions; research and development rarely progress in a straight line, and innovation is fraught with unintended consequences. Small wonder, then, if the outcomes of biomedical investment often differ from what was promised, with implications for society that go far beyond simply delivering new means of prevention and treatment.

The difficulty in foreseeing exactly how local, national, and global biomedical initiatives will pan out is not a reason for resigned acceptance of whatever those programmes deliver, however. On the contrary, it underscores the need for close and continuing societal engagement and involvement with biomedicine, from the initial formulation of promising research programmes, right through to the design and implementation of new health interventions, and at all stages in between. Efforts to promote such engagement and involvement are still in their infancy, and it is far from clear what forms they might ultimately take and how they might influence innovation trajectories and health experiences. Yet, if biomedicine is to develop in ways that respond most effectively to wider societal needs, it is imperative that such efforts continue in a spirit of open-minded collaboration and informed critical reflection.

As growing numbers of scientists and clinicians, funders and policy makers recognise, researchers in the social sciences and humanities possess knowledge and skills that equip them to play an invaluable role in informing, facilitating, and critiquing these endeavours ( Pickersgill *et al*., 2018). Hence, for instance, the Wellcome Trust’s own commitment to fund research in the humanities and social sciences, “to bring new perspectives and ways of thinking to the historical, ethical and cultural contexts in which medical science takes place” ( Wellcome Trust, 2010, p. 17). Likewise, the increasingly common practice of inviting social scientists and humanities scholars to participate in technoscientific projects, especially but not only in biomedicine, with the aim of securing improved governance and social acceptability. Yet, such collaborations bring novel challenges, as participants from within and across different disciplines try to work out their respective roles in the business of shaping technoscience ( Balmer *et al*., 2016; Callard & Fitzgerald, 2015; Frickel *et al.*, 2016). Still, the need for such collaborations is paramount: biomedicine is reshaping our lives in multifaceted ways, through interrelated transformations of knowledge and practice, research and care, institutions and forms of governance, and individual and collective identities. Only by working across disciplinary boundaries, and with patients and publics, can we begin to comprehend these transformations in all their complexity, in ways that will enable us to engage constructively as well as critically with the dynamic relationships between science, public health, and healthcare; the shifting geographies of global, national, and local institutions; and the lived experiences of individuals and social groups around the world.

## Transformations of biomedicine, self and society

There can be no simple prescription for how to build such collaborations, but we can at least begin to map the kinds of questions and issues they might most fruitfully seek to address. Social scientific and humanities research into the socio-cultural system that is biomedicine has already produced a wealth of insights into the nature and meaning of biomedical science and healthcare in societies. Drawing on such work, we can identify five interconnecting domains of individual and societal experience which provide fertile ground on which to cultivate new inter- and transdisciplinary enquiry. We now reflect on these and some of the issues they bring into focus.

### Disease

Contemporary developments in biomedicine not only challenge long-accepted ideas about the nature of particular diseases; they also raise more general questions about just what counts as disease. This has enormous implications also for the organisation and delivery of health care, and for the sociotechnical organisation of biomedical science, it will have profound impact on individuals’ experience of health and illness. In the case of cancer, for instance, increasingly ‘personalised’ understandings of tumour aetiology are fragmenting existing classificatory schemes and exacerbating clinical uncertainty, even as they offer the hope of new and more effective treatments ( Keating & Cambrosio, 2011; Kerr & Cunningham-Burley, 2015). At the same time, population research into biomarkers in cancer and other conditions is leading to new categories of pre-disease risk, bringing new classes of ‘patients-in-waiting’ under medical management ( Aronowitz, 2015; Timmermans & Buchbinder, 2010). In psychiatry, the objectivity of established clinical tools for diagnosing mental disorders is being questioned by research funders, and there are calls for fundamental revision of existing diagnostic categories in the hope of driving therapeutic innovation for mental ill-health ( Pickersgill, 2014). Multi-dimensional approaches to population studies of experiences associated with psychiatric disorder are complicating understandings of the boundaries between normality and pathology. In society more widely, debates over disability, normalcy, ‘naturalness’ and enhancement further problematize concepts of disease and therapy, across a range of contexts spanning mental, physical and reproductive health and biomedicine ( Nuffield Council on Bioethics, 2015). Meanwhile increasing costs, earlier preventive interventions and an ever-widening range of ways in which individuals seek to access biomedical technologies only add to the uncertainties about what counts as health, illness, and disease, and who benefits or suffers from those designations.

Together, these developments raise fundamental questions about the nature of disease, about who has the epistemic authority to label it as such, and about the normative and political significance of the disease concept itself. Biomedical research alone cannot answer these questions; indeed, as the relationship between research and clinical practice becomes increasingly blurred, the issues only become more complex. Close engagement with social science and humanities research, as well as patients and their families, will help us all to navigate the fraught borderlands between health, illness, and disease in ways that best address the needs of individuals and populations.

### Bodies

As a key site of biomedical intervention, the human body is increasingly chemically regulated, technologically augmented, and digitally rendered. The diverse connections between individuals and bodies and body-parts is intensely mediated by technical, social, and legal instruments ( Crawford, 2014; Flear *et al*., 2013; Hoeyer, 2013; Mol, 2008; Quigley, 2018). In the case of emerging innovations such as 3D bioprinting of replacement organs, for instance, this raises questions around justice, social stratification, and regulation ( Vermeulen *et al.*, 2017). Other technoscientific developments in genetics and genomics, pharmaceuticals, nanotechnology, and biotechnological devices have implications for individual experience and collective representations of embodiment. The changing relationship between subjectivity and bodily identity and integrity potentially destabilises established ontologies of human beings. New vulnerabilities are created ( Oudshoorn, 2016), contributing to a unique form of ‘biomedical nemesis’ ( Haddow, forthcoming; cf. Illich, 1974). Simultaneously, practices such as ‘biohacking’ and the ‘DIY Bio’ movement challenge who should be permitted to create hybrid artificial-organic bodies and techno-scientific identities, pushing the limits of current medical and legal understandings of control and responsibility. The increased datafication of the human body is arguably reducing aspects of the physical human form to inexhaustible datasets ( Parry & Greenhough, 2017), signalling the prospect of data flowing freely within and beyond the biomedical sphere, amplifying social and ethical concerns around the privacy and security of individuals, groups, and populations ( Pink & Lanzeni, 2018).

Research in the social sciences and humanities provides a rich legacy of concepts and methods for understanding and accessing human embodied experience, challenging the mind-body dualism once inherent to ‘Western’ medicine. Contemporary research is beginning to shed light on how the possibilities of bodily repair, replacement and regeneration affect the ways in which individuals experience themselves as embodied beings. Fundamental questions about why changes to the materiality of the body alter subjectivity are brought into sharper relief ( Haddow *et al*., 2015). We need such forms of interdisciplinary research to apprehend fully the complexities of radical amelioration and enhancement ( Pickersgill & Hogle, 2015). Careful and critical thinking with scientific researchers and clinical practitioners about how biomedical expertise and experts themselves (actively or otherwise) are informing and structuring shifting meanings and, indeed, what counts as embodied experience, is required.

### Global movements and institutions

Accelerating global movements of people, goods, data, and capital have led to new geographies, both of health, illness, and disability, and of technoscientific practice ( Bozorgmehr, 2010; Keane, 1998). Despite commitments to globalisation and increased investments worldwide, the uneven spatial and socioeconomic distribution of disease and ill-health continues ( Clinton & Sridhar, 2017). With an exponential increase in funding for global health initiatives, and the creation of a global health technocracy, local concerns are increasingly over-written by transnational flows of ideas and resources, especially in low and middle income countries ( Adams, 2016). Health priorities and practices are typically constructed through universalistic discourses of science and economics ( Sridhar, 2011), ascribing value to individuals and their bodies and calculating the impact of disease in ways that elide local meanings, needs, and values ( Anand *et al.*, 2004; Leach & Tedros, 2014).

Models such as the Global Burden of Disease project funded by the Bill and Melinda Gates Foundation mediate the traffic between local and global understandings of disease. We need to critically interrogate the relevance and limitations of such models for priority-setting in health policy. At the same time, there is a too common tendency to frame financially poorer nations solely as beneficiaries of healthcare knowledge, as opposed to producers of it ( White *et al.*, 2014). Without attention to the different roles that low income countries can, should, and do play with respect to the production of biomedical and other health-related knowledges and the actualization of new biotechnologies, we may fail to confront the problems of global scientific as well as health justice ( Chan *et al*., 2017). Novel, spatial analyses of contemporary biomedicine must be attentive to the consequences of its glocalization ( Robertson, 2012)—the ways in which biomedical ideas, actions, and artefacts not only circulate internationally, but are simultaneously localised and particularised. In so doing, they also need to break away from path-dependent normative and empirical approaches and foci that all too often rehearse the colonial tropes they ostensibly aim to undermine. Collaborations between biomedicine, global health, social science, and humanities combining local and global contexts, that also engage populations across different nations, will help propel a more radical and transformative agenda for global public health.

### Law

Law and legal processes and their associated institutions are present throughout biomedicine, from intellectual property regimes to regulation of clinical research to protection of individual and collective patients’ rights. All too often, law is perceived to place barriers along the route from science to health; for instance, by obstructing research or by creating artificial boundaries between different jurisdictions or sectors of research ( Academy of Medical Sciences, 2011). However, such perceptions fail to recognise that law also plays a constitutive role in biomedical research and practice ( Cloatre & Pickersgill, 2014). This is not least through helping to establish norms and standards for scientific knowledge production, material and intellectual exchange, constructive participation, and effective ethical review that lead to social value ( Ganguli-Mitra *et al*., 2017). For instance, regulatory impasse may be overcome through interdisciplinary, iterative engagement with scientific communities to design and deliver adaptable systems of research governance ( Laurie *et al.*, 2012).

A deep focus must now be paid to how law functions as an intrinsic element of biomedical culture, including its role in the processes of developing frameworks for pre-emptive regulation that enable the emergence and consolidation of socially acceptable and accountable biomedical science, as well as in enabling the stewardship of biomedical researchers through regulatory environments ( Laurie *et al*., 2018). Examining how regulatory stakeholders construct legality around their actions and with what consequences ( Richards, 2015) demands a blending of social scientific and humanities insights to understand how regulators and regulated actors (including scientists and clinicians) co-produce adaptable regulatory practices while raising questions about the letter of the law itself ( Stephens *et al*., 2011). Whether, where, when, and how law decelerates biomedical development and application, or acts as an accelerator of multiple stakeholder-desired change, demands subtle and careful attention to the multiple effects of law and legal processes. Put briefly, we need far deeper understandings of law as a lived experience within biomedicine than we possess at present.

### Science-society engagements

Participation and partnerships are increasingly mobilised in the production of biomedical research, the development of health-related policy, and health care delivery ( Involve, 2005). From individual patient involvement through to population-wide engagement, multiple sites of participation characterise the nexus between biomedicine, healthcare organisations, social groups, and individual experiences of health and illness. Politicians, scientists, clinicians, and wider publics can have complex relationships with the processes, practices, and outcomes of engagements. For instance, different forms and instances of engagement can be viewed variously as an asset and barrier to policy development, health research, and service delivery across clinical and public health. Matters are further complicated by the increasing blurring of patienthood and participation in (for example) data-intensive population studies, where patients become at once producers of commercially valuable data as well as consumers of healthcare ( Prainsack, 2017). Funders and research regulators may also intervene to shape the relationship between research, engagement, and participation; for example, by requiring public and patient involvement as an essential element of research strategies and projects.

Social scientists have led a critical understanding of public engagement with respect to science and its governance ( Irwin & Michael, 2003; Jasanoff, 2003; Kelty & Panofsky, 2014), and many have been key to shaping the institutional contexts and individual experiences of engagement with biomedicine ( Aitken *et al.*, 2016; Aitken *et al*., 2018; Haddow *et al.*, 2007). Parallel developments in bioethical thinking have tracked evolving paradigms of research ethics and the changing role of participants with respect to science ( Emanuel & Grady, 2007). Understanding and shaping effective participation requires both social scientific appreciation of the expertise that non-specialists can bring to shaping science and its regulation ( Cunningham-Burley, 2006; Kerr *et al*., 2007), and bioethical reflection on how best to reconceptualise the normative dimensions of participation ( Chan & Harris, 2009; Chan *et al*., 2011). Explorations of how publics are currently interpolated within biomedicine, and with what consequences, are necessary in their own right ( TNS, 2015). We also have to ask difficult and potentially uncomfortable questions about whether, how, and when different groups should engage to maximise the public good.

## Conclusion

The need to comprehend and interrogate contemporary transformations in knowledge, health, and experience is vital. Inequalities persist despite enormous advances in prevention and treatments; new technologies of research and care are troubling accepted ideas about the human body, health and disease; and the expansion and proliferation of citizen engagement and partnerships bring both opportunities and challenges for biomedical science and healthcare. These pressing issues demand imaginative and innovative responses. As a considerable body of scholarship implies ( Cunningham-Burley, 2006; Irwin & Michael, 2003; Kerr *et al*., 2007; Webster, 2002), close engagement between diverse communities is required to shape, direct, and, indeed, personalise biomedical research in ways that deliver the greatest social benefit. Research within and between the medical social sciences and humanities is vital. So, too, are novel partnerships between scholars in those fields with scientists, clinicians, and policy-makers. Researchers across academic disciplines could do much more to work effectively together in order to understand the complexities inherent in biomedical science, and to promote the kinds of social as well as technical change that will deliver equitable promotion of health and wellbeing locally, nationally, and globally. They also need to engage enthusiastically, deliberatively and openly with wider publics and civic society. Ultimately, we need a new social contract between biomedicine and society that better serves the aim of improved health and well-being for all. If we are to achieve this, it will not be enough to challenge one other from the security of our own disciplinary perspectives, whichever they are, nor simply to try to reconcile our different disciplinary commitments. Together we need to rethink biomedicine from the ground up. 

## Data availability

No data is associated with this article.

## References

[ref-5] Academy of Medical Sciences: A New Pathway for the Regulation and Governance of Health Research. *Academy of Medical Sciences.* 2011 Reference Source

[ref-1] Adams V: What is critical global health? *Med Anthro Theory.* 2016;3:186–197. 10.17157/mat.3.2.429

[ref-2] Aitken M Cunningham-Burley S Pagliari C: Moving from trust to trustworthiness: Experiences of public engagement in the Scottish Health Informatics Programme. *Sci Public Policy.* 2016;43(5):713–723. 10.1093/scipol/scv075 28066123 PMC5210028

[ref-3] Aitken M Tully M Porteous C: International consensus statement on public involvement and engagement with data-intensive health research. *Int J Pop Data Sci.* 2018;3(4). 10.23889/ijpds.v3i4.837 PMC8142968 34095528

[ref-4] Anand S Peters F Sen A: Public Health, Ethics, and Equity. Oxford: Oxford University Press.2004 Reference Source

[ref-7] Aronowitz R: Risky Medicine: Our Quest to Cure Fear and Uncertainty. Chicago: University of Chicago Press.2015 Reference Source

[ref-8] Balmer A Calvert J Marris C: Taking roles in interdisciplinary collaborations: reflections on working in post-ELSI spaces in the UK synthetic biology Community. *Sci Tech Studies.* 2016;28(3):3–25. Reference Source

[ref-9] Brown N Michael M: A sociology of expectations: *retrospecting prospects and prospecting retrospects*. *Technol Anal Strateg Manage.* 2003;15(1):3–18. 10.1080/0953732032000046024

[ref-10] Bozorgmehr K: Rethinking the ‘global’ in global health: a dialectic approach. *Global Health.* 2010;6:19. 10.1186/1744-8603-6-19 21029401 PMC2987787

[ref-11] Callard F Fitzgerald D: Rethinking Interdisciplinarity across the Social Sciences and Neurosciences. New York: Palgrave.2015. 10.1057/9781137407962 26726692

[ref-12] Chan S Harris J: Free riders and pious sons--why science research remains obligatory. *Bioethics.* 2009;23(3):161– 71. 10.1111/j.1467-8519.2008.00648.x 18445091 PMC3579232

[ref-13] Chan S Palacios-González C De Jesús Medina Arellano M: Mitochondrial Replacement Techniques, Scientific Tourism, and the Global Politics of Science. *Hastings Cent Rep.* 2017;47(5):7–9. 10.1002/hast.763 28940343 PMC6052433

[ref-16] Chan S Zee Y Jayson G: Risky research and participants’ interests: the case of phase 2c clinical trials. *Clinical Ethics.* 2011;6(2):91–96. 10.1258/ce.2011.011019

[ref-14] Clinton C Sridhar D: Governing Global Health: Who Runs the World and Why?Oxford: Oxford University Press.2017 Reference Source

[ref-15] Cloatre E Pickersgill M: Knowledge, Technology and Law. Abingdon: Routledge.2014 10.4324/9780203797600

[ref-17] Crawford CS: Phantom Limb: Amputation, Embodiment, and Prosthetic Technology. New York: New York University Press.2014 10.18574/nyu/9780814789285.001.0001

[ref-18] Cunningham-Burley S: Public knowledge and public trust. *Community Genet.* 2006;9(3):204–10. 10.1159/000092658 16741351

[ref-19] Emanuel E Grady C: Four paradigms of clinical research and research oversight. *Camb Q Healthc Ethics.* 2007;16(1):82–96. 10.1017/S0963180107070090 17345970

[ref-20] Feiler T Gaitzkell K Maughan T(eds.): Personalized Medicine: The Promise, the Hype and the Pitfalls. *Special Issue, The New Bioethics: A Multidisciplinary Journal of Biotechnology and the Body.* 2017;23(1–12). 10.1080/20502877.2017.1314895 28517985

[ref-21] Flear ML Farrell AM Hervey TK(eds.): European Law and New Health Technologies. Oxford: Oxford University Press.2013 10.1093/acprof:oso/9780199659210.001.0001

[ref-22] Frickel S Albert M Prainsack B, (eds.): Investigating Interdisciplinary Collaboration: Theory and Practice across Disciplines. New Brunswick: Rutgers University Press.2016 Reference Source 27854405

[ref-23] Ganguli‐Mitra A Dove ES Laurie GT: Reconfiguring Social Value in Health Research Through the Lens of Liminality. *Bioethics.* 2017;31(2):87–96. 10.1111/bioe.12324 28060429 PMC5244658

[ref-26] Haddow G: Embodiment and Everyday Cyborgs: Technologies of Altered Subjectivity. Manchester: Manchester University Press. (forthcoming). Reference Source 34236780

[ref-25] Haddow G King E Kunkler I: Cyborgs in the Everyday: Masculinity and Biosensing Prostate Cancer. *Sci Cult (Lond).* 2015;24(4):484–506. 10.1080/09505431.2015.1063597 27335534 PMC4894087

[ref-24] Haddow G Laurie G Cunningham-Burley S: Tackling community concerns about commercialisation and genetic research: a modest interdisciplinary proposal. *Soc Sci Med.* 2007;64(2):272–282. 10.1016/j.socscimed.2006.08.028 17050056

[ref-27] Hoeyer K: Exchanging Human Bodily Material: Rethinking Bodies and Markets. Dordrecht: Springer.2013 10.1007/978-94-007-5264-1

[ref-28] Illich I: Medical Nemesis. London: Calder & Boyars. *Lancet.* 1974;303(7863):918–921. 10.1016/S0140-6736(74)90361-4 4133432

[ref-29] Involve: People and Participation. London: Involve.2005 Reference Source

[ref-30] Irwin A Michael M: Science, Social Theory and Public Knowledge. Maidenhead: Open University Press.2003 Reference Source

[ref-31] Jasanoff S: Technologies of humility: citizen participation in governing science. *Minerva.* 2003;41(3):223–44. 10.1023/A:1025557512320

[ref-32] Keane C: Globality and constructions of world health. *Med Anthropol Q.* 1998;12(2):226–240. 10.1525/maq.1998.12.2.226 9627924

[ref-33] Keating P Cambrosio A: Cancer on Trial: Oncology as a New Style of Practice. Chicago: University of Chicago Press.2011 Reference Source

[ref-34] Kelty C Panofsky A: Disentangling public participation in science and biomedicine. *Genome Med.* 2014;6(1):8. 10.1186/gm525 24479693 PMC3979030

[ref-35] Kerr A Cunningham-Burley S Tutton R: Shifting subject positions: experts and lay people in public dialogue. *Soc Stud Sci.* 2007;37:385–411. 10.1177/0306312706068492

[ref-70] Kerr A Cunningham-Burley S: Embodied innovation and regulation of medical technoscience: transformations in cancer patienthood. *Law Innov Technol.* 2015;7(2):187–205. 10.1080/17579961.2015.1106103 27996062 PMC5139616

[ref-36] Laurie GT Dove ES Ganguli-Mitra A: Charting Regulatory Stewardship in Health Research: Making the Invisible Visible. *Cam Q Healthc Ethics.* 2018;27(2):333–347. 10.1017/S0963180117000664 29509122 PMC5848747

[ref-37] Laurie G Harmon S Arzuaga F: Foresighting futures: law, new technologies, and the challenges of regulating for uncertainty. *Law Innov Tech.* 2012;4(1):1–33. 10.5235/175799612800650626

[ref-38] Leach M Tedros M: Epidemics and the politics of knowledge: contested narratives in Egypt's H1N1 response. *Med Anthropol.* 2014;33(3):240–54. 10.1080/01459740.2013.842565 24761977

[ref-39] Mol AM: The Logic of Care: Health and the Problem of Patient Choice. Abingdon: Routledge.2008 Reference Source

[ref-40] Nuffield Council on Bioethics: Ideas about Naturalness in Public and Political Debates about Science, Technology and Medicine. Analysis paper. November 2015.2015 Reference Source

[ref-41] Oudshoorn N: The vulnerability of cyborgs: the case of ICD shocks. *Sci Technol Human Values.* 2016;41(5):767–792. 10.1177/0162243916633755

[ref-42] Parry B Greenhough B: Bioinformation.Cambridge: Polity.2017 Reference Source

[ref-43] Pickersgill MD: Debating DSM-5: diagnosis and the sociology of critique. *J Med Ethics.* 2014;40(8):521–25. 10.1136/medethics-2013-101762 24327375 PMC4112449

[ref-45] Pickersgill M Chan S Haddow G: The social sciences, humanities, and health. *Lancet.* 2018;391(10129):1462–1463. 10.1016/S0140-6736(18)30669-X 29676265

[ref-44] Pickersgill M Hogle L: Enhancement, ethics and society: towards an empirical research agenda for the medical humanities and social sciences. *Med Humanit.* 2015;41(2):136–142. 10.1136/medhum-2015-010718 26260624 PMC4717454

[ref-46] Pink S Lanzeni D: Future anthropology ethics and datafication: temporality and responsibility in research. *Soc Media Society.* 2018;4(2):1–9. 10.1177/2056305118768298

[ref-47] Prainsack B: Personalized Medicine: Empowered Patients in the 21 ^st^ Century?New York: New York University Press.2017 Reference Source

[ref-48] Quigley M: Self-Ownership, Property Rights, and the Human Body: A Legal and Philosophical Analysis. Cambridge: Cambridge University Press.2018 Reference Source

[ref-49] Richards S: Unearthing bureaucratic legal consciousness: government officials’ legal identification and moral ideals. *Int J Law Context.* 2015;11(3):299–319. 10.1017/S1744552315000166

[ref-50] Robertson R: Globalisation or glocalisation? *Journal of International Communication.* 2012;18(2):191–208. 10.1080/13216597.2012.709925

[ref-51] Rose N: The Politics of Life Itself: Biomedicine, Power, and Subjectivity in the Twenty-First Century.Princeton: Princeton University Press.2007 Reference Source

[ref-53] Sridhar D: Health policy: from the clinical to the economic gaze. *Lancet.* 2011;378(9807):1909 10.1016/S0140-6736(11)61829-1

[ref-54] Stephens N Atkinson P Glasner P: Documenting the doable and doing the documented: bridging strategies at the UK Stem Cell Bank. *Soc Stud Sci.* 2011;41(6):791–813. 10.1177/0306312711423306 22400420

[ref-55] Timmermans S Buchbinder M: Patients-in-waiting: Living between sickness and health in the genomics era. *J Health Soc Behav.* 2010;51(4):408–23. 10.1177/0022146510386794 21131618

[ref-56] TNS: Factors Affecting Public Engagement by Researchers: A Study on Behalf of A Consortium of UK Public Research Funders. TNS: London.2015 Reference Source

[ref-57] Vermeulen N Haddow G Seymour T: 3D bioprint me: a socioethical view of bioprinting human organs and tissues. *J Med Ethics.* 2017;43(9):618–624. 10.1136/medethics-2015-103347 28320774 PMC5827711

[ref-58] Webster A: Innovative health technologies and the social: redefining health, medicine and the body. *Curr Sociol.* 2002;50(3):443–457. 10.1177/0011392102050003009

[ref-59] Wellcome Trust: Strategic Plan 2010-2020: Extraordinary Opportunities. London: Wellcome Trust.2010 Reference Source

[ref-60] White R Jain S Giurgi-Oncu C: Counterflows for mental well-being: what high-income countries can learn from low and middle-income countries. *Int Review Psychiatry.* 2014;26(5):602–606. 10.3109/09540261.2014.939578 25343638

