# Supplementary material for: Biomedicine, self and society: An agenda for collaboration and engagement
Source: Wellcome Open Res. 2019 Jan 23;4:9. [Version 1] doi: 10.12688/wellcomeopenres.15043.1 (PMC6367656; doi:10.12688/wellcomeopenres.15043.1)
Supplement: Peer review history

# Peer review: Referee response for version 1

**DOI:** 10.21956/wellcomeopenres.16412.r34661

**Published:** 2019-02-06

**Authors:** Barbara Prainsack

**Affiliations:** 1 Department of Political Science, University of Vienna, Vienna, Austria; 2 Department of Global Health & Social Medicine, King's College London, London, UK

This is a very thoughtful and timely contribution to the debate on interdisciplinary collaboration in the health domain. The authors emphasise the important role of "authentic collaborations across and between the biomedical sciences, humanities and social sciences, and wider society” to create new knowledge in biomedicine. They then identify and discuss five domains which they argue are of particular relevance for such collaborations (and particularly impacted by them in turn). 

 I consider this Letter a timely and substantive intervention into debates, funding programmes, and policies shaping knowledge production in this field. In the following section I will share a few thoughts that the authors may, or may not, take up in their further work on this topic.

1. Underpinning the argument of the authors is the assumption that relevant biomedical knowledge can no longer be disciplined into clearly delineated domains of expertise and practice. This is a profound point that could be made more explicit. What are the reasons for this? The authors mention “fundamental transformations in the generation and application of knowledge [that] challenge our understandings and experiences of health, illness, and disease as well as the organisation of research and care”. But what are these transformations? For example, how are we to understand the attempts by researchers and policy makers to bring research and healthcare (e.g. in precision medicine), individual- and population-level data and interventions (e.g. in precision public health), and the social and the biological (e.g. in epigenetics) closer together? What types of cross-disciplinary and cross-domain collaborations made this possible, and what types of collaborations do these new convergences require? 
2. The names of the five domains that the authors identify - disease, bodies, global movements and institutions, law, and science-society engagements - are labeled in traditional biomedical and global health terminology. To reflect the spirit of the cross-domain and cross-disciplinary collaboration that the authors highlight in their Letter, it may be better to focus not only on disease but also on illness, not only on bodies but also on people, and on the collaboration between global and local institutions.
3. As critical scholars have pointed out, much of the literature conceptualises collaboration as *transactions* between bounded independent entities. An alternative ontology would be to conceive collaboration as *interactions* between entities that are related to each other through shared interests, stakes, (and, in case of human actors, also shared identities). When reading the Letter I was wondering what ontology underpins the authors’ call for more “authentic" collaboration. What makes collaborations authentic? What types of actors and relations does authentic collaboration require? 
4. The authors’ point, on p4, that “there is a too common tendency to frame financially poorer nations solely as beneficiaries of healthcare knowledge, as opposed to producers of it” is a very important one, and one that perhaps deserves even more emphasis and elaboration. How can collaboration have a positive impact on changing these dominant frames? I also wonder how collecting data from people in low- and middle income countries (LMIC) would be considered by the authors: Does this count as healthcare knowledge creation on the side of people in LMIC countries, or does it increase the new social division between data givers and data takers? Or both?
5. As a reader of this Letter I would love to hear what the authors consider good examples for the types of collaborations that they call for. Perhaps it may be possible to name a few examples in the concluding section, or to give an example in each of the five domains.

I have read this submission. I believe that I have an appropriate level of expertise to confirm that it is of an acceptable scientific standard.

# Peer review: Referee response for version 1

**DOI:** 10.21956/wellcomeopenres.16412.r34663

**Published:** 2019-01-31

**Authors:** Andrew Webster

**Affiliations:** 1 Science and Technology Studies Unit (SATSU) , University of York, York, UK

This is a timely and important Open Letter inasmuch as current developments in diverse areas of biomedicine, and the life sciences more generally, disturb conventional understandings of the body/medicine/society relationship. The article rightly points out that as boundaries - of the body, of disease, of care, of responsibility and so on - become more blurred new forms of  collaboration across the biomedical sciences, social sciences and humanities is required. New complexities call for new collaborative endeavour.

 The various sections provide very useful, concise overviews of how these disturbances shape different domains of biomedicine, with a solid body of supportive references to support the argument. Each of these domains pose specific challenges to biomedicine if it is to be socially robust, that is one that combines innovation with accountability. The debate in recent years over 'responsible research and innovation' is clearly of relevance here.

 It is then extremely important to show how in practice this interdisciplinary collaboration might be made possible, and thereby move from a position of critique (key though that is to any engagement) to one of co-production. Here there is some unevenness in the article, with perhaps most clear recommendations on what might be involved seen in the concluding observations in the 'Global movements and institutions' and 'Law' sections. 

 To unpack this comment a little more, we can note that in many ways the increasing differentiation of disease classification systems and the subsequent disruption of the normal/abnormal boundary is to be welcomed by the social sciences/humanities who have long seen hard borders as problematic (as noted especially in the section on 'Bodies' in regard to multiple meanings of 'embodiment').  At the same time, how social science then helps people  - patients/populations/clinicians/biomedical researchers - to create more valuable and socially robust ways of handling disease and illness becomes an ever greater  challenge as a result, especially if we are to participate in the co-production of meaning and practice. Complexity of practices and how these can be managed by patients, clinical staff and carers, for example, become of central importance (eg in regard to the complexities of end-of-life care). So here social science needs to address how to handle three interrelated but discrete elements - the complexity of phenomena, the personal difficulties experienced by people and uncertainty over possible futures.

 The challenge for those who take home the very important message of this article is how to find sites where this form of knowledge is possible and what a new community of practice combining disciplines would look like. The article concludes by a call to 'rethink biomedicine from the ground up'. Given the programmatic nature of this piece presumably we need to also rethink social science/humanities 'from the ground up' too. Perhaps the best place to do this is through identifying trans-disciplinary sites where new methodologies can be co-created for building a shared understanding, especially when seeking to shape policy and practice ^1^. The challenge in particular is to somehow bridge between what has been called 'critical interdisciplinary research' and 'instrumental interdisciplinary' research ^2^^-^^3^ - that is, challenging conventional understanding in a constructive way.  The authors themselves have shown how this is possible in their own prior work.  Perhaps an indication of the challenges they faced and how they were resolved would have helped to point the reader, new to this debate, in the right (interdisciplinary) direction.

I have read this submission. I believe that I have an appropriate level of expertise to confirm that it is of an acceptable scientific standard.

## References

[1] WebsterA: Crossing Boundaries Social Science in the Policy Room. *Science, Technology, & Human Values*.2007;32(4) : 10.1177/0162243907301004 458-478 10.1177/0162243907301004

[2] KleinJT: The taxonomy of interdisciplinarity. *Frodeman, R., Klein, J.T., Mitcham, C. (Eds.), Oxford Handbook of Interdisciplinarity. Oxford: Oxford University Press*.2010;

[3] HuutoniemiKKleinJBruunHHukkinenJ: Analyzing interdisciplinarity: Typology and indicators. *Research Policy*.2010;39(1) : 10.1016/j.respol.2009.09.011 79-88 10.1016/j.respol.2009.09.011
